# Homologous and Heterologous Expression of Delta(12)-Desaturase in *Mucor circinelloides* Enhanced the Production of Linolenic Acid

**DOI:** 10.3390/molecules27175511

**Published:** 2022-08-27

**Authors:** Junhuan Yang, Xiuwen Wang, Hassan Mohamed, Shaoqi Li, Chen Wu, Wenyue Shi, Futing Xue, Sergio López-García, Yuanda Song

**Affiliations:** 1 Colin Ratledge Center for Microbial Lipids, School of Agricultural Engineering and Food Science, Shandong University of Technology, Zibo 255000, China; 2 Department of Food Sciences, College of Food Science and Engineering, Lingnan Normal University, Zhanjiang 524048, China; 3 Department of Botany and Microbiology, Faculty of Science, Al-Azhar University, Assiut 71524, Egypt; 4 Department of Genetics and Microbiology (Associated Unit to IQFR-CSIC), Faculty of Biology, University of Murcia, 3100 Murcia, Spain

**Keywords:** homologous expression, heterologous expression, *Mucor circinelloides*, delta-12 desaturase, lipid accumulation, linolenic acid production

## Abstract

Linolenic acid (LA) is gaining more interest within the scientific community. This is because it has a potential medical role in reducing the risk of inflammation, carcinogenesis, atherosclerosis and diabetes and is a valuable nutraceutical for human health. The oleaginous fungus *Mucor circinelloides* produces a high lipid content (36%), including valuable polyunsaturated fatty acids (PUFAs). However, the critical step in which oleic acid (OA) is converted into LA is not efficient at supplying enough substrates for PUFA synthesis. Hence, we propose a method to increase LA production based on genetic engineering. The overexpression of the Δ12-desaturase gene from *M. circinelloides* and *Mortierella alpina* increased the LA content and improved the lipid accumulation (from 14.9% to 21.6% in the Δ12-desaturase gene of the *M. circinelloides* overexpressing strain (Mc-D12MC) and from 14.9% to 18.7% in the Δ12-desaturase gene of *M. alpina* overexpressing strain (Mc-D12MA)). Additionally, the up-regulated expression levels of these genes targeted the genes involved in NADPH production, implying that the elevated Δ12-desaturase gene may function as a critical regulator of NADPH and lipid synthesis in *M. circinelloides*. This study provides the first evidence to support the design of metabolic engineering related to LA and PUFA production in *M. circinelloides* for potential industrial applications.

## 1. Introduction

Polyunsaturated fatty acids (PUFAs) have essential structural and functional roles in the human body. They are responsible for cell membrane fluidity and protein activity or interactions with other molecules [1]. In addition, PUFAs such as γ-linolenic acid (GLA, C18:3), arachidonic acid (ARA, C20:4), eicosapentaenoic acid (EPA, C20:5), and docosahexaenoic acid (DHA, C22:6) have been used as food additives to fortify specific foods [1,2]. Mammals cannot synthesize linolenic acid (LA). Therefore, LA belongs to the group of essential fatty acids (EFAs) [3]. One of the most promising EFA sources is microbial lipids, which were proposed to be high-value PUFAs produced during the past two decades [4]. Additionally, some microbial produced fatty acid methyl esters (FAMEs) can be used as biofuels and biodiesel [5].

For the fatty acid biosynthesis pathway, oleaginous microorganisms require acetyl-CoA as a substrate generated via cytosol citrate cleavage and catalyzed by ACL (ATP: citrate lyase) [6]. In microorganisms, the citrate transported from the mitochondria to the cytoplasm increases the cytoplasmic citrate concentration, resulting in lipid accumulation [7]. The second essential co-factor agent is an NADPH agent acting as a reducing agent provided by the reactions in the pentose phosphate pathway, malate degradation, and cytosolic NADP^+^-dependent isocitrate dehydrogenase (NADP^+^: ICDH) [8]. PUFAs are initially synthetized from stearic acid (C18:0). Oleic acid (OA, C18:1) is converted from C18:0 by Δ9-desaturase and then desaturated into linoleic acid (LA, C18:2) by Δ12-desaturase. LA is the specific substrate of Δ6-desaturase and Δ15-desaturase used to generate GLA and α-linolenic acid (ALA). Subsequent elongation and desaturation lead to the generation of long-chain PUFAs, such as EPA and DHA [3]. Thus, LA is the base for the synthesis of PUFAs. Compared with plant seed oils (the traditional resource of LA), microbial lipids have the advantages of a fast-growing rate, simple culture conditions, and controllable products [9].

*Mucor circinelloides* was used as a commercial strain (in which the lipid content is 25% of the cell dry weight (CDW) for GLA production in the 1980s [2]. Recently, the lipid overproducing strain *M. circinelloides* WJ11, isolated from soil, presented 36% of its own CDW [10], utilizing a wide range of carbon sources (i.e., glucose [11], xylose [12], and glycerol [13]).

*Mucor circinelloides* can be genetically modified to increase the lipid content and the LA yield [14,15,16]. Lamers and co-workers [3] increased the LA content (from 5.6% to 23.3% and 22.7%) in *Schwanniomyces occidentalis* by homologously and heterologously expressing FAD2. Subsequently, Δ12-fatty acid desaturase was successfully expressed in *Rhodotorula toruloides* and improved the final titer of LA to 1.3 g/L [17]. LA is a precursor and limiting factor for PUFA biosynthesis in *M. circinelloides*, since there is only one Δ12-desaturase gene in its genome, and the LA content is approximately 15 % of the total lipids [10]. Thus, to improve the LA productivity, we overexpressed the Δ12-desaturase genes from *M. alpina* and *M. circinelloides* [10]. To investigate the underlying mechanism of Δ12-desaturase in LA production and lipid accumulation, the growth patterns, lipid contents, fatty acid composition, and gene expressing levels of the engineered strains were analyzed. This is the first report to study the impact of the homologous and heterologous expressions of Δ12-desaturase and investigate the possibility of improving LA production by genetic engineering in *M. circinelloides*. The present study has the potential to improve the commercial value of microbial lipids and could provide a novel and sustainable source of LA-rich lipids.

## 2. Materials and Methods

### 2.1. Strains, Transformation, and Fermentation Conditions

The Δ12-desaturase genes from *M. alpina* (ATCC32222) and *M. circinelloides* WJ11 (CCTCC No. M 2014424) were amplified from the cDNA libraries of these two strains [18]. *M. circinelloides* MU760, an uracil and leucine auxotrophic strain of *M. circinelloides* WJ11, was used as the recipient strain for Δ12-desaturase genes. During the genetic manipulations, transformants were selected in the minimal culture medium MMC and in MMC with the uracil medium, as previously described [19]. The Kendrick and Ratledge (K and R) medium was used in the fermentation process for lipid production [20]. For the analysis of the lipid production, approximately 10^6^ spores of each strain were added to 100 mL of K & R medium in 500 mL baffled flasks and cultured in a shaker at 28 °C for 1 day, and then transferred all the cultures into a fermenter that contained 1 L K & R medium. The fermentation conditions were set at a stirring speed of 600 rpm, aeration of 1.0 min^−1^, temperature maintained at 28 °C, and pH adjusted to 6.0 using 1 mol/L NaOH.

The *Escherichia coli* Top 10 (for cloning and plasmid storage) was cultured in an LB medium supplemented with ampicillin or kanamycin (100 mg/L) when necessary [21].

### 2.2. The Construction of Δ12-Desaturase Gene Overexpression Plasmids and Transformants

The pMAT2075 plasmid containing a selectable maker (*pyrF*) and a strong promoter (*pzrt1*) surrounded by 1 kb up-/down-stream *CarRP* sequences was used to insert the target genes. The Δ12-desaturase gene fragments were amplified from the cDNA libraries of *M. alpina* and *M. circinelloides* by the primers D12MA-F/R and D12MC-F/R (Supplementary Materials, Table S1), respectively. Amplified fragments were inserted into the linearized vector pMAT2075 digested with *XhoI* to generate pMAT2075-D12MA and pMAT2075-D12MC using the One Step cloning kit. The transformants were constructed as previously described [22,23] and grown in MMC for several vegetative cycles to obtain the heterokaryons for both Δ12-desaturase gene overexpression strains.

### 2.3. Biochemical Analysis of the Gene-Overexpressing Transformants during the Fermentation Process

To analyze the impacts of Δ12-desaturase gene overexpression on the fermentation process of *M. circinelloides*, biomass, glucose, and nitrogen consumption were monitored during cultivation in a 1 L bioreactor for 4 days. The supernatant of the fermentation samples was collected at 6, 12, 24, 36, 48, 60, 72, 84, and 96 h. Glucose concentration was measured by using a glucose oxidase electrode biosensor (SBA-40E, Institute of Biology, Shandong Academy of Sciences, China) [24]. Nitrogen depletion was determined by using the indophenol method [25].

Fungal mycelia were harvested by a Buchner funnel filtration, washed three times by distilled H_2_O, and freeze-dried for biomass and lipid analysis. For lipid and fatty acid analysis, 10 mg of freeze-dried mycelium were used for extraction using chloroform/methanol (2:1, *v*/*v*). Pentadecanoic acid (C15:0) was added to the extract as an internal standard, and the mixture was methylated by using 10% HCL/methanol (*w*/*w*). The fatty acid methyl esters (FAMEs) were analyzed by gas chromatography (GC) equipped with a 30 m × 0.32 mm DB-Waxetr column [26]. The GC program for FAME determination was set as follows: 120 °C for 3 min, ramp to 200 °C at 5 °C/min, then ramp to 220 °C at 4 °C/min, and hold 2 min.

### 2.4. Genomic DNA Extraction, RNA Preparation, and Quantitative Reverse Transcription-PCR Analysis

Transformants’ genomic DNA was extracted using the DNA Quick Plant System kit (Tiangen Biotech Co., Ltd., Beijing, China). Total RNA was extracted with Trizol and reverse transcribed to cDNA library using Evo M-MLV RT Mix Kit with gDNA Clean for qPCR (Accurate Biotech Co., Ltd., Changsha, China). Quantitative reverse transcription-PCR analysis (RT-qPCR) was performed using SYBR Green Realtime PCR Master Mix in a LightCycler 96 (Rotkreuz, Switzerland). Actin was used as an internal reference gene, and the 2^−ΔΔCt^ method was applied to calculate the gene expression levels. The primers used in this study are listed in the Supplementary Materials (Supplementary Materials, Table S1).

### 2.5. Statistical Analysis

All of the experimental results were calculated as the mean data from three independent experiments, and the data were presented as mean ± SD. The student’s *t*-test (SPSS 21.0, IBM, Armonk, NY, U.S.A.) was used for statistical analysis of the data, and differences were considered statistically significant at *p* < 0.05.

## 3. Results

### 3.1. Generation of M. Circinelloide Δ12-Desaturase Gene Overexpression Transformants by Genetic Engineering

**Figure 1:**
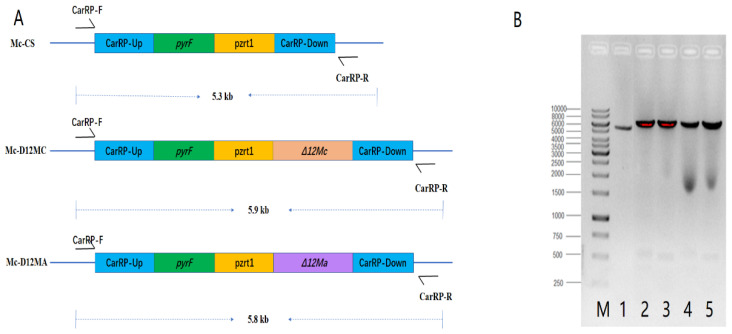
Generation of *Δ12Mc* and *Δ12Ma* gene overexpression transformants. (**A**) Genomic structure of the control strain (Mc-CS) and gene overexpression transformants (Mc-D12MC and Mc-D12MA). (**B**) The PCR verification of the recombination strains: Mc-CS (lane 1), two transformants of Mc-D12MC (lane 2, 3) and two transformants of Mc-D12MA (lane 4, 5) with the CarRP-F/R primers.

Based on the cDNA sequences of the Δ12-desaturase genes from *M. alpina* and *M. circinelloides*, two genes (*Δ12Mc* and *Δ12Ma*) were cloned into the expression vector pMAT2075 individually and generated gene overexpressing plasmids pMAT2075-D12MC and pMAT2075-D12MA. The transformants (named Mc-D12MC and Mc-D12MA) were constructed by homologous recombination of the linearized fragments that contained the selectable marker *pyrF* gene, the target gene, and the genes flanking 1 kb up- and down-stream from the *carRP* gene. The control strain Mc-CS was generated by the fragments in pMAT2075 (Figure 1A). The PCR analysis was used to confirm that the target genes were successfully integrated into the genome of the transformants by using primers (CarRP-F and R) (Supplementary Materials, Table S1). Bands approximately 5.9 kb (Mc-D12MC) and 5.8 kb (Mc-D12MA) in size showed the positive integration events, whereas a band at about 5.3 kb was amplified from the control strain Mc-CS (Figure 1B). These results confirmed that the target genes were successfully integrated into the genome of *M. circinelloides* WJ11, separately.

### 3.2. Expression Levels of Δ12-Desaturase Gene from M. Alpina and M. Circinelloides in Mc-D12MA and Mc-D12MC

**Figure 2:**
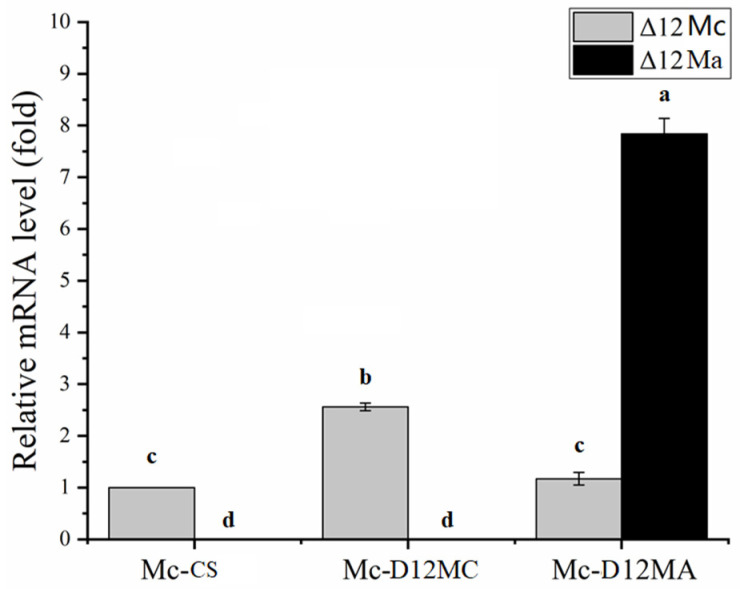
Transcription levels of *Δ12Mc* and *Δ12Ma* genes in the transformants Mc-D12MC, Mc-D12MA, and Mc-CS. The mRNA in the transformants at 24 h was extracted and measured by RT-qPCR. The expression levels of *Δ12Mc* (gray bars) and *Δ12Ma* (black bar) were quantified by rt-D12Mc-F/R and rt-D12Ma-F/R primers, separately. The values are the mean of three independent determinations. The error bars represent the standard error. The different letters present the statistically significant differences between the values (*p* < 0.05).

The transcriptional levels of the Δ12-desaturase gene from *M. alpina* and *M. circinelloides* were analyzed in the transformants at 24 h by RT-qPCR using correlative primers (Supplementary Materials, Table S1). As shown in Figure 2, the expression level of the *Δ12Mc* gene in the overexpressing strain (Mc-D12MC) was 2.5-fold higher than that in the control strain, which suggested that *Δ12Mc* was successfully overexpressed in Mc-D12MC. Meanwhile, *Δ12Ma* only showed a significant expression level in Mc-D12MA, indicating that *Δ12Ma* was successfully overexpressed in *M. circinelloides* without affecting the native *Δ12Mc* expression pattern.

### 3.3. Cell Growth and Lipid Accumulation in Mc-D12MC and Mc-D12MA Strains

**Figure 3:**
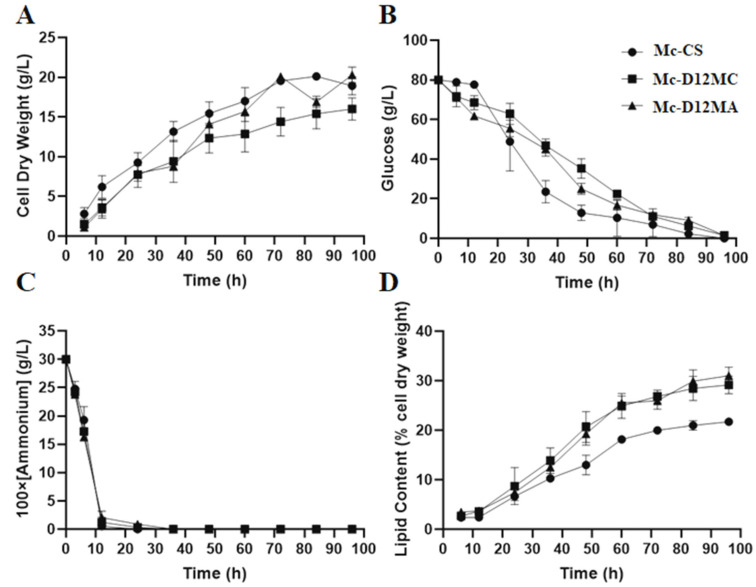
Fermentation analysis of the Δ12-desaturase gene-overexpressing strains. (**A**) Cell dry weight (CDW), (**B**) glucose concentration, (**C**) ammonium concentration, and (**D**) lipid contents in Mc-CS (circle), Mc-D12MC (square), and Mc-D12MA (triangle) grown in a 1 L fermenter were measured. The values are the mean of three independent experiments. The error bars represent the standard error of the mean.

Cell growth, glucose, and NH_4_^+^ concentration in the culture supernatant and lipid contents in the *Δ12Ma* and *Δ12Mc* overexpressing transformants were detected, and the results are displayed in Figure 3. The growth patterns of these three strains were similar (Figure 3A): the biomass of three strains increased with rapid speed before 48 h, thereafter, showing a slower growth state. Compared to Mc-CS (the control strain), the Mc-D12MC showed a lower biomass growth, whereas Mc-D12MA was similar to the control after 48 h of growth (Figure 3A). The glucose and the nitrogen consumption rates of these three strains were similar (Figure 3B,C). Once the nitrogen source was depleted, the lipids began to accumulate rapidly. Manipulation of the Δ12-desaturase gene significantly affected the fatty acid synthesis in *M. circinelloides* (Figure 3D). The fatty acid content of the Mc-D12MC strain was increased by about 21.8 % (from 22 % to 26.8 %), while the fatty acid content of the Mc-D12MA strain was increased to 25.9 %, an increase of 17.7 % compared with Mc-CS.

### 3.4. Overexpression of Δ12-Desaturase Gene Affected the Levels of Fatty Acid in the Transformants

**Figure 4:**
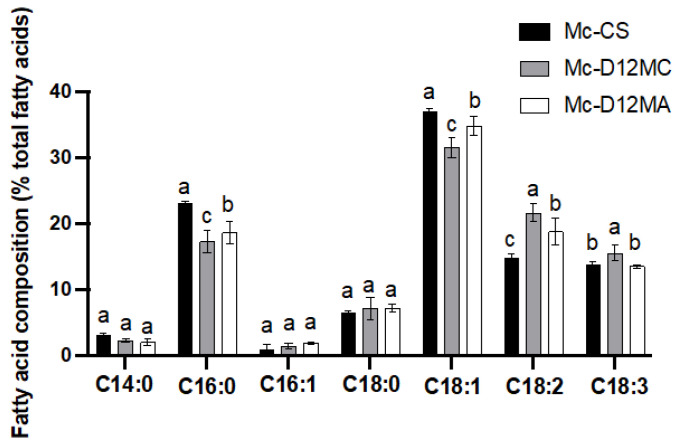
Fatty acid composition of lipids in Mc-D12MC, Mc-D12MA, and the control strains. The values are the mean of three independent experiments. The error bars represent the standard error of the mean. The different letters present the statistically significant differences between the values (*p* < 0.05).

The fatty acid profiles of these transformants elucidated that the overexpression of Δ12-desaturase genes have a significant influence on the composition of fatty acids. Compared with the control strain Mc-CS (Figure 4), the LA (C18:2) significantly increased in Mc-D12MC and Mc-D12MA and the palmitic acid (C16:0) and OA (C18:1) were remarkedly decreased in both transformants. Therefore, the overexpression of the Δ12-desaturase gene had an important role in the conversion from OA (C18:1) to LA (C18:2) and affected the other fatty acid metabolism.

### 3.5. The Transcription Level of Key Genes for Fatty Acid Desaturation in Mc-D12MC and Mc-D12MA

**Figure 5:**
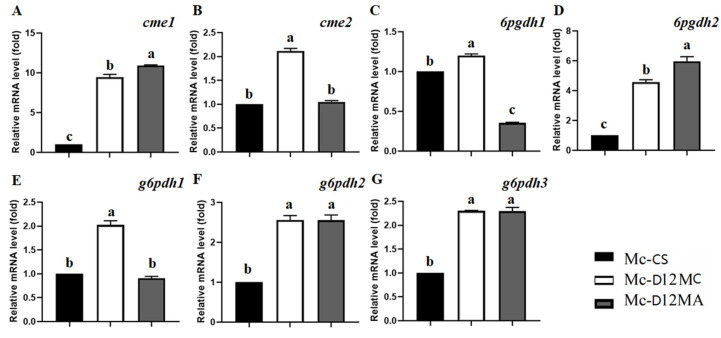
Expression levels of several key genes involved in NADPH biosynthesis in Mc-D12MC, Mc-D12MA, and the control strain. The mRNA expressing levels were determined at 24 h of fermentation by RT-qPCR. *cme*, cytosolic malic enzyme; *g6pdh*, glucose-6-phosphate dehydrogenase; *6pgdh*, 6phosphogluconate dehydrogenase. The values are the mean of three independent experiments. The error bars represent the standard error of the mean. The different superscripts present the statistically significant differences between the values (*p* < 0.05).

The growth results showed that drastic changes had taken place in response to the manipulation of Δ12-desaturase gene in transformants. To better understand the transcriptional response of fatty acid desaturation occurring, we extracted the total RNA and analyzed the transcript levels of several genes in these strains (the control, Mc-D12MC, and Mc-D12MA) during the fermentation process. The genes (*cme1*, gene ID: scaffold00036.12; *cme2*, and gene ID: scaffold00049.37) encoded for the malic enzyme in cytoplasm (cME) were chosen for analysis since the cME was assumed to be important for the generation of the reducing power in oleaginous microorganisms [27,28,29]. The results showed that the *cme1* expression level was greatly increased in both gene overexpressing transformants, whereas *cme2* was only up-regulated in Mc-D12MC (Figure 5). Meanwhile, because 6-phosphogluconate dehydrogenase (6PGDH) and glucose-6-phosphate dehydrogenase (G6PDH) are two key enzymes in the pentose phosphate pathway (PPP) in providing the reducing power for lipid synthesis [8,16,30], the genes encoded for these two proteins were analyzed. There were two genes encoding for 6PGDH (*6pgdh1*, gene ID: scaffold00113.18; *6pgdh2*, gene ID: scaffold00142.5) and three genes encoding for G6PDH (*g6pdh1*, gene ID: scaffold00053.31; *g6pdh2*, gene ID: scaffold00034.42; and *g6pdh3*, gene ID: scaffold00081.31) in *M. circinelloides*. As shown in Figure 5, the expression levels of *6pgdh2*, *g6pdh2*, and *g6pdh3* were up-regulated significantly in Mc-D12MC and Mc-D12MA.

## 4. Discussion

Oleaginous microorganisms can accumulate lipids that account for 20–80% of the CDW, depending on the species and cultures. Especially, when the nitrogen source is depleted, the excess carbon source is converted into a fatty acid and stored in tri-acylglycerol forms [31]. The oleaginous microbes are investigated globally as an alternative sustainable feedstock to plant oils for potential biodiesel production [32]. These oil-rich microorganisms can accumulate up to 70% of their CDW, with a predominance of specific fatty acid, mainly OA and L [33]. One of the most promising oleaginous microorganisms is *M. circinelloides*, which was generally recognized as the model oleaginous organism for the lipid accumulation and oil production. In addition, the genetic tools for filamentous fungal metabolic engineering have been established, including the single and double auxotroph strains, multiple gene overexpression, and deletion systems that allowed for the rewriting of the pathways involved in fatty acid synthesis and improvements in the engineering capacity for producing tailored fatty acids in *M. circinelloides* [14]. However, the lipids produced by *M. circinelloides* WJ11 contained a low LA level (around 15% in total fatty acids) [34]. Thus, the reaction converting OA to LA catalyzed by Δ12-desaturase might be a critical step in PUFA synthesis, since LA is the essential precursor for a series of desaturation and elongation reactions in the PUFA biosynthesis pathways [3]. Therefore, in this study, we overexpressed the Δ12-desaturease from *M. alpina* and *M. circinelloides* to investigate possible ways to overcome obstacles in obtaining LA-rich microbial lipids.

The phenotypical results suggested that the Δ12-desaturease gene from both *M. alpina* and *M. circinelloides* shared physiological functions in the *M. circinelloides* WJ11. We also observed that overexpression of the Δ12-desaturease gene from *M. alpina* and *M. circinelloides* in the WJ11 strain caused an increase in lipid production and suppression of cell growth in the nitrogen limited broth in the early fermentation stage (Figure 3A,D). Considering unsaturated fatty acids such as LA plays a vital role in ameliorating cell membrane fluidity and integrity [35]. Thus, an explanation was proposed that LA increment in cells negatively affects transformants’ growth. The lower cell growth also reduced the glucose-consumption speed (Figure 3b).

OA is the substrate for LA synthesis. Hence, we expected that the overexpression of Δ12-desaturease would further increase the lipid accumulation and LA production. The present study observed significantly higher LA contents in both gene overexpressing transformants in the present study (Figure 4). Compared with the control strain, the LA percentages of the Mc-D12MC and Mc-D12MA strains were increased by 45% and 25.5%, respectively. Our results are entirely consistent with previous research reporting that the overexpression of Δ12-desaturase in the oleaginous yeast *Rhodotorula toruloides* increased the production of LA-rich lipids from 14.0% in the wild-type strain to 28.1% in the Δ12-desaturease gene homologous expressing strain ^17^.

To further investigate the underlying mechanism of Δ12-desaturase in improving LA production and lipid accumulation, the present work has studied the genes generating NADPH, the redox cofactor, which is a critical factor for the biosynthesis of valuable lipids in oleaginous cells [8]. Therefore, we analyzed the expression levels of genes using RT-qPCR. Known genes that encode malic enzymes, glucose-6-phosphate dehydrogenase, and 6-phosphogluconate dehydrogenase involved in NADPH production were examined (Figure 5). The analysis of mRNA levels showed that at least one of the gene copies encoding these enzymes was significantly increased in both transformants. Moreover, the transcription levels of *cme*, *g6pdh*, and *6pgdh* were all up-regulated in the Mc-D12MC and Mc-D12MA strains. Therefore, the increased NADPH supply may be a reason for the higher total unsaturated fatty acid content of the total fatty acids in the Mc-D12MC strain (70.0%) than that in Mc-D12MA (68.9% of the total fatty acids). Considering the significant increase in LA for the total fatty acid and the lipids in transformants cells, we can hypothesize that the overexpression of the Δ12-desaturease could increase the activities of key enzymes in the NADPH synthesis pathway and further increase LA production and lipid accumulation.

## 5. Conclusions

We demonstrated the development of a genetic manipulation strategy for improving LA production, associated with increased NADPH availability resulting from the overexpression of the Δ12-desaturease gene in *M. alpina* and *M. circinelloides*. The data presented an improvement in the LA production and specific lipid accumulation. Therefore, Δ12-desaturease gene overexpression in *M. circinelloides* can be used to solve the blockage that occurs during high-value fatty acid synthesis. However, further work is required to better understand the mechanisms associated with promising lipid production in organisms to soon be used in the pharmaceutical industry.

## Data Availability

Not applicable.

## Supplementary Materials

The following are available online at https://www.mdpi.com/article/10.3390/molecules27175511/s1, Table S1: Primers and their sequences used in this study.

## Sample Availability

All data are included in the article/Supplementary Material, further inquiries can be directed to the corresponding author(s).

## References

[1] Bellou S., Triantaphyllidou I.E., Aggeli D., Elazzazy A.M., Baeshen M.N., Aggelis G. (2016). Microbial oils as food additives: Recent approaches for improving microbial oil production and its polyunsaturated fatty acid content. Curr. Opin. Biotechnol..

[2] Kikukawa H., Watanabe K., Kishino S., Takeuchi M., Ando A., Izumi Y., Sakuradani E. (2022). Recent trends in the field of lipid engineering. J. Biosci. Bioeng..

[3] Lamers D., Visscher B., Weusthuis R.A., Francke C., Wijffels R.H., Lokman C. (2019). Overexpression of delta-12 desaturase in the yeast *Schwanniomyces occidentalis* enhances the production of linoleic acid. Bioresour. Technol..

[4] Tavares S., Grotkjaer T., Obsen T., Haslam R.P., Napier J.A., Gunnarsson N. (2011). Metabolic engineering of *Saccharomyces cerevisiae* for Production of Eicosapentaenoic Acid, Using a Novel Δ5-Desaturase from *Paramecium tetraurelia*. Appl. Environ. Microb..

[5] Singh G., Jeyaseelan C., Bandyopadhyay K.K., Paul D. (2018). Comparative analysis of biodiesel produced by acidic transesterification of lipid extracted from oleaginous yeast *Rhodosporidium toruloides*. 3 Biotech.

[6] Carsanba E., Papanikolaou S., Erten H. (2018). Production of oils and fats by oleaginous microorganisms with an emphasis given to the potential of the nonconventional yeast *Yarrowia lipolytica*. Crit. Rev. Biotechnol..

[7] Yang J., Li S., Kabir Khan M.A., Garre V., Vongsangnak W., Song Y. (2019). Increased lipid accumulation in *Mucor circinelloides* by overexpression of mitochondrial citrate transporter genes. Ind. Eng. Chem. Res..

[8] Ratledge C. (2014). The role of malic enzyme as the provider of NADPH in oleaginous microorganisms: A reappraisal and unsolved problems. Biotechnol. Lett..

[9] Ledesma-Amaro R., Dulermo R., Niehus X., Nicaud J.M. (2016). Combining metabolic engineering and process optimization to improve production and secretion of fatty acids. Metab. Eng..

[10] Tang X., Zhao L., Chen H., Chen Y.Q., Chen W., Song Y., Ratledge C. (2015). Complete genome sequence of a high lipid-producing strain of *Mucor circinelloides* WJ11 and comparative genome analysis with a low lipid-producing strain CBS 277.49. PLoS ONE.

[11] Ratledge C., Wynn J.P. (2002). The biochemistry and molecular biology of lipid accumulation in oleaginous microorganisms. Adv. Appl. Microbiol..

[12] Zhang Y., Song Y. (2021). Lipid accumulation by xylose metabolism engineered *Mucor circinelloides* strains on corn straw hydrolysate. Appl. Biochem. Biotechnol..

[13] Zan X., Sun J., Chu L., Cui F., Huo S., Song Y., Koffas M.A.G. (2021). Improved glucose and xylose co-utilization by overexpression of xylose isomerase and/or xylulokinase genes in oleaginous fungus Mucor circinelloides. Appl. Microbiol. Biotechnol..

[14] Yang J., Canovas-Marquez J.T., Li P., Li S., Niu J., Wang X., Nazir Y., Lopez-Garcia S., Garre V., Song Y. (2021). Deletion of plasma membrane malate transporters increased lipid accumulation in the oleaginous fungus *Mucor circinelloides* WJ11. J. Agric. Food Chem..

[15] Zhang Y., Wang Y., Yang J., Yang W., Wang X., Wu C., Song Y. (2022). Improved γ-Linolenic acid production from cellulose in *Mucor circinelloides* via coexpression of cellobiohydrolase and delta-6 desaturase. J. Agric. Food Chem..

[16] Zhao L., Tang X., Luan X., Chen H., Chen Y.Q., Chen W., Song Y., Ratledge C. (2015). Role of pentose phosphate pathway in lipid accumulation of oleaginous fungus Mucor circinelloides. RSC Adv..

[17] Wu C.C., Ohashi T., Kajiura H., Sato Y., Misaki R., Honda K., Limtong S., Fujiyama K. (2021). Functional characterization and overexpression of Delta12-desaturase in the oleaginous yeast Rhodotorula toruloides for production of linoleic acid-rich lipids. J. Biosci. Bioeng..

[18] Ye C., Xu N., Chen H., Chen Y.Q., Chen W., Liu L. (2015). Reconstruction and analysis of a genome-scale metabolic model of the oleaginous fungus *Mortierella alpina*. BMC Syst. Biol..

[19] Nicolas F.E., de Haro J.P., Torres-Martinez S., Ruiz-Vazquez R.M. (2007). Mutants defective in a Mucor circinelloides dicer-like gene are not compromised in siRNA silencing but display developmental defects. Fungal. Genet. Biol..

[20] Kendrick A., Ratledge C. (1992). Lipid formation in the oleaginous mould Entomophthora exitalis grown in continuous culture: Effects of growth rate, temperature and dissolved oxygen tension on polyunsaturated fatty acids. Appl. Microbiol. Biotechnol..

[21] Zan X., Tang X., Chu L., Song Y. (2018). Dual functions of lip6 and its regulation of lipid metabolism in the oleaginous fungus *Mucor circinelloides*. J. Agric. Food Chem..

[22] Garre V., Barredo J.L., Iturriaga E.A. (2015). Transformation of *Mucor circinelloides f. lusitanicus* Protoplasts. Genet. Transform. Syst. Fungi.

[23] Gutierrez A., Lopez-Garcia S., Garre V. (2011). High reliability transformation of the basal fungus Mucor circinelloides by electroporation. J. Microbiol. Methods.

[24] Sun M.-L., Madzak C., Liu H.-H., Song P., Ren L.-J., Huang H., Ji X.-J. (2017). Engineering Yarrowia lipolytica for efficient γ-linolenic acid production. Biochem. Eng. J..

[25] Chaney A.L., Marbach E.P. (1962). Modified reagents for determination of urea and ammonia. Clin. Chem..

[26] Wang X., Mohamed H., Bao Y., Wu C., Shi W., Song Y., Yang J. (2021). Heterologous expression of two malate transporters from an oleaginous fungus *Mucor circinelloides* improved the lipid accumulation in *Mucor lusitanicus*. Front. Microbiol..

[27] Song Y., Wynn J.P., Li Y., Grantham D., Ratledge C. (2001). A pre-genetic study of the isoforms of malic enzyme associated with lipid accumulation in *Mucor circinelloides*. Microbiology.

[28] Hao G., Chen H., Du K., Huang X., Song Y., Gu Z., Wang L., Zhang H., Chen W., Chen Y.Q. (2014). Increased fatty acid unsaturation and production of arachidonic acid by homologous over-expression of the mitochondrial malic enzyme in *Mortierella alpina*. Biotechnol. Lett..

[29] Zhang Y., Liu Q., Li P., Wang Y., Li S., Gao M., Song Y. (2021). Enhanced lipid production by addition of malic acid in fermentation of recombinant Mucor circinelloides Mc-MT-2. Sci. Rep..

[30] Rodriguez-Frometa R.A., Gutierrez A., Torres-Martinez S., Garre V. (2013). Malic enzyme activity is not the only bottleneck for lipid accumulation in the oleaginous fungus *Mucor circinelloides*. Appl. Microbiol. Biotechnol..

[31] Mhlongo S.I., Ezeokoli O.T., Roopnarain A., Ndaba B., Sekoai P.T., Habimana O., Pohl C.H. (2021). The potential of single-cell oils derived from filamentous fungi as alternative feedstock sources for biodiesel production. Front. Microbiol..

[32] Meeuwse P., Sanders J.P.M., Tramper J., Rinzema A. (2013). Lipids from yeasts and fungi: Tomorrow’s source of biodiesel?. Biofuels Bioprod. Biorefining.

[33] Srinivasan N., Thangavelu K., Sekar A., Sanjeev B., Uthandi S. (2021). Aspergillus caespitosus ASEF14, an oleaginous fungus as a potential candidate for biodiesel production using sago processing wastewater (SWW). Microb. Cell Fact..

[34] Tang X., Chen H., Chen Y.Q., Chen W., Garre V., Song Y., Ratledge C. (2015). Comparison of Biochemical Activities between High and Low Lipid-Producing Strains of Mucor circinelloides: An Explanation for the High Oleaginicity of Strain WJ11. PLoS ONE.

[35] Henderson C.M., Block D.E. (2014). Examining the role of membrane lipid composition in determining the ethanol tolerance of Saccharomyces cerevisiae. Appl. Environ. Microbiol..

