# Supplementary material for: Homologous and Heterologous Expression of Delta(12)-Desaturase in Mucor circinelloides Enhanced the Production of Linolenic Acid
Source: Molecules. 2022 Aug 27;27(17):5511. doi: 10.3390/molecules27175511 (PMC9457725; doi:10.3390/molecules27175511)
Supplement: Supplementary file 1 [file molecules-27-05511-s001.zip › molecules-1876918-supplementary.pdf]

Supplementary Table S1. Primers and their sequences used in this study

Primers	Sequences (5'-3')	annotation
D12MC-F	TACAAAATAACTAAATAATGCTCGAGATGGC AACCAAGAGAAACG	Simple cloning of *D12MC* gene (*Xho I*)
D12MC-R	GAGCTCATCTGCTTGATCTCGAGACTCTTAGT TCTTAAAG	Simple cloning of *D12MC* gene (*Xho I*)
D12MA-F	ACAAAATAACTAAACTCGAGTAATGATGGCA CCTCCCAACACTATTG	Simple cloning of *D12MA* gene (*Xho I*)
D12MA-R	CATGAGCTCATCTGCTTGATCTCGAGACTCTT ACTTCTTGAAAAAGAC	Simple cloning of *D12MA* gene (*Xho I*)
CarRP-F	GATAAGCATAAACCAGATCTGC	Amplification of fragment on the genome
CarRP-R	GTATCTGACATAGTCGAGCT TC	Amplification of fragment on the genome
rt-D12MC-F	CAGGTCTTTGTCCCCTCTACTCG	RT-qPCR for *D12MC* gene located in genome
rt-D12MC-R	ATCTTGACCAGAGACATTGGTG	RT-qPCR for *D12MC* gene located in genome
rt-D12MA-F	ATGTTCCACGGCATTGTTCACAC	RT-qPCR for *D12MA* gene located in genome
rt-D12MA-R	CAAATCGGCACTCCCGGAAC	RT-qPCR for *D12MA* gene located in genome
rt-53.31-F	ATTGTTTCACAGAGGAAGAGACTTTCC	RT-qPCR for *g6pd1* gene located in genome
rt-53.31-R	TGTTATCAATGTTGGAACGGTTCCAG	RT-qPCR for *g6pd1* gene located in genome
rt-34.42-F	TGTCATGCAAAATCATTTGCTGCAG	RT-qPCR for *g6pd2* gene located in genome
rt-34.42-R	GACATACTGTCCCAAGAGAGAATCTT CC	RT-qPCR for *g6pd2* gene located in genome
rt-81.31-F	TGCACTCTTCACTGAAAATGAGATTT ATCG	RT-qPCR for *g6pd3* gene located in genome

rt-81.31-R	TTATCAATATAGGTGCGATCCCATGC	RT-qPCR for *g6pd3* gene located in genome
rt-113.18-F	GATGGTTCACAACGGTATTGAATACGGA	RT-qPCR for *6pgd1* gene located in genome
rt-113.18-R	CGATCAAGAAAGAATCCAATTCACCCTTG	RT-qPCR for *6pgd1* gene located in genome
rt-142.5-F	GCACAACGGTATTGAGTACGGC	RT-qPCR for *6pgd2* gene located in genome
rt-142.5-R	CCCTTATTCCATTCATCAAAGACATC	RT-qPCR for *6pgd2* gene located in genome
rt-36.12-F	TGATTCCAAGGGTCTTGTTACTACTACACG	RT-qPCR for *cme1* gene located in
